# Growth hormone-releasing hormone (GHRH) and its agonists inhibit hepatic and tumoral secretion of IGF-1

**DOI:** 10.18632/oncotarget.25676

**Published:** 2018-06-19

**Authors:** Tengjiao Cui, Andrew V. Schally

**Affiliations:** ^1^ Endocrine, Polypeptide and Cancer Institute, Veterans Affairs Medical Center, Miami, FL, USA; ^2^ Department of Medicine, Divisions of Hematology, Oncology and Endocrinology, University of Miami, Miami, FL, USA; ^3^ Department of Pathology, University of Miami, Miami, FL, USA; ^4^ Sylvester Comprehensive Cancer Center, Miller School of Medicine, University of Miami, Miami, FL, USA

**Keywords:** IGF-1, GHRH agonists, hepatocytes, tumor, JAK2/STAT5 signaling

## Abstract

The role of hypothalamic growth hormone-releasing hormone (GHRH) in the release of growth hormone (GH) from the pituitary is well established. However, direct effects of GHRH and its agonistic analogs on extra-pituitary cells and tissues have not been completely elucidated. In the present study, we first demonstrated that human and rat hepatocytes express receptors for GHRH. We then showed that GHRH(1-29)NH_**2**_ and GHRH agonist, MR-409, downregulated mRNA levels for IGF-1 in human cancer cell lines and inhibited IGF-1 secretion *in vitro* when these cancer lines were exposed to rhGH. Another GHRH agonist, MR-356, lowered serum IGF-l and inhibited tumor growth in nude mice bearing xenografted NCI-N87 human stomach cancers. GHRH(1-29)NH_**2**_ and MR-409 also suppressed the expression of mRNA for IGF-1 and IGF-2 in rat and human hepatocytes, decreased the secretion of IGF-1 *in vitro* from rat hepatocytes stimulated with rhGH, and lowered serum IGF-l levels in hypophysectomized rats injected with rhGH. Vasoactive intestinal peptide had no effect on the release of IGF-1 from the hepatocytes. Treatment of C57BL/6 mice with MR-409 reduced serum levels of IGF-l from days 1 to 5. These results show that GHRH and its agonists can, by a direct action, inhibit the secretion of IGF-1 from the liver and from tumors. The inhibitory effect of GHRH appears to be mediated by the GHRH receptor (GHRH-R) and GH receptor (GHR), with the involvement of JAK2/STAT5 pathways. Further studies are required to investigate the possible physiopathological role of GHRH in the control of secretion of IGF-1.

## INTRODUCTION

During the past years, our group reported the synthesis of multiple antagonistic analogs of growth hormone releasing hormone (GHRH) and the biological evaluation of their inhibitory effects on growth of various tumors [1, 2]. We have also synthesized powerful agonistic analogs of GHRH [3] and demonstrated that in rodents, they improve cardiac function after experimental myocardial infarction and reduce cardiac hypertrophy [4-6], stimulate the proliferation and regeneration of pancreatic islet cells [7, 8], and accelerate wound healing [9]. Although for many years the sole role of GHRH was thought to be the regulation of the release of growth hormone (GH) from the pituitary, these findings show that GHRH and its analogs can exert direct effects on extra-pituitary cells/tissues.

Insulin-like growth factor 1 (IGF-l), plays an essential physiological role on the growth and development, [10-13]. The functions of IGF-1 in the development and progression of cancers are also well documented. IGF-1 has been shown to stimulate cancer cell proliferation, inhibit apoptosis [14, 15] and facilitate metastasis by promoting cell migration and invasion [16]. Most circulating IGF-1 is produced by the liver, following the stimulation with pituitary GH [17]. High levels of circulating IGF-1 have been linked to an increased risk of neoplasia and poor prognosis in many clinical studies [18, 19]. Various studies have been carried out on secretion of IGF-l in physiological, clinical and pathological conditions [20-25], but to the best of our knowledge the involvement of a neurohumoral component in these situations has not been previously identified. This paper describes our investigations of the effects of GHRH and its agonists on the synthesis and release of IGF-l in normal liver tissue and in tumors.

## RESULTS

### Human and rat hepatocytes express GHRH receptor

**Figure 1:**
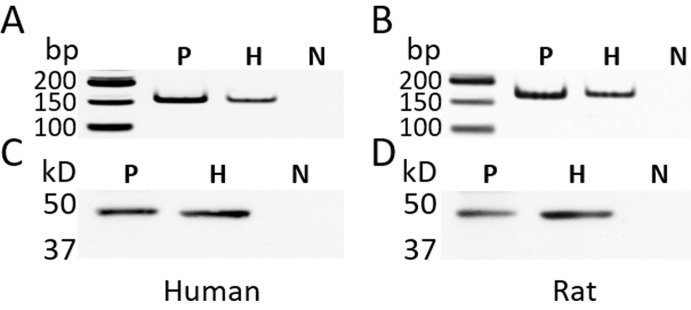
The expression of GHRH receptor (GHRH-R) in the primary human hepatocytes and rat hepatocytes **A.**, **B.** The PCR-based amplification of a fragment from GHRH-R cDNA. The size of the PCR product from the human cDNA (A) was 155 bp and from rat cDNA (B) 164 bp. **C.**, **D.** Western blots using a rabbit polyclonal antibody (Abcam 76263) against human GHRH-R (C) and rat GHRH-R (D). P: human or rat pituitary, positive control; H: human or rat hepatocytes; N: negative control; The reaction without cDNA was used for the negative control of PCR. In the Western blots, the primary antibody against GHRH-R was replaced by normal rabbit IgG. bp: base pair; kD: kilodalton.

The presence of GHRH receptor in primary human and rat hepatocytes was determined by a PCR-based method and Western blots. Human or rat pituitaries were used as positive controls. PCR primers, (F) GATGAGAGTGCCTGTCTACAAGCA, (R) TCTGAGCTGAAGTGAGAGAAGAAATC, were designed to target a unique region between exon 2 and exon 3 of mRNA for human GHRH-R (Genebank: NM_000823). The PCR primers, (F) CCTTCCAGGGTTTTGTTGTTG, (R) GGTGAGCACCTTCACTCTCGAT, were used to target mRNA for rat GHRH-R (Genebank: NM_012850). The PCR products amplified from the cDNA of human/rat hepatocytes and their respective pituitary control exhibited the expected size (Figure 1A, 1B). The specificity of the results of PCR was further verified by DNA sequencing (data not shown). The expression of GHRH receptor at the protein level was determined by Western blots. The GHRH-R antibody recognized a band of 47 kD (Figure 1C, 1D) in the pituitary and the hepatocytes from both human and rats, which matched the calculated size of the unglycosylated pituitary type GHRH receptor. Both the PCR and Western blot findings confirmed the presence of GHRH receptor in primary human and rat hepatocytes.

### GHRH(1-29)NH_2_ and its agonist, MR-409 suppress the expression of mRNA for IGF-1 and IGF-2 in human and rat hepatocytes

**Figure 2:**
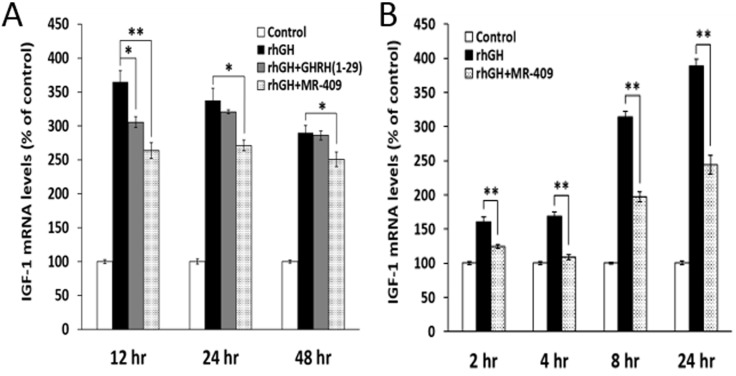
GHRH(1-29)NH_2_ and its analog, MR-409, inhibit the expression of IGF-1 in primary hepatocytes Human **A.** or rat **B.** hepatocytes were treated with either recombinant human growth hormone (rhGH) alone (0.5 µg/ml for human hepatocytes and 1 µg/ml for rat hepatocytes) or a combination of rhGH with 1 µM GHRH(1-29)NH_2_ or MR-409. The RNAs were isolated at selected times and the levels of mRNA for IGF-1 were determined by quantitative RT-PCR. (Error bars indicate ±SEM; *p < 0.05, **p < 0.01).

Direct effects of GHRH(1-29)NH_2_ and its analog, MR-409, on the expression of IGF-1 in primary hepatocytes was investigated *in vitro*. Human or rat hepatocytes were treated with rhGH alone or in combination with GHRH(1-29)NH_2_ or MR-409. As shown in the Figure 2A, in human hepatocytes, both GHRH(1-29)NH_2_ and MR-409 inhibited the IGF-1 expression induced by treatment with rhGH (0.5 µg/ml). The peptides by themselves, however, shows no effect (Supplementary Figure 1A). In the human hepatocytes, IGF-1 expression stimulated by rhGH was decreased by16.2±2.2% (*p* < 0.05), at 12 hours in the presence of 1 µM GHRH(1-29)NH_2_, but the decrease at 24 hours (5.1±0.8%) and 48 hours (1.5±2.3%) was not significant. In the cells treated with rhGH and 1 µM MR-409, mRNA for IGF-1 decreased by 27.7±3.2% (*p* < 0.01) at 12 hours, by 19.7±2.3% (*p* < 0.05) at 24 hours, and by 13.6±3.7% (*p* < 0.05) at 48 hours, compared to cells treated with rhGH alone. In rat hepatocytes, an inhibition was observed as early as 2 hours after treatment by 1 µM MR-409 (Figure 2B). The mRNA for IGF-1 decreased significantly by 22.3±1.8% (*p* < 0.01) at 2 hours, 35.5±2.1% (*p* < 0.01) at 4 hours, 37.1±2.2% (*p* < 0.01) at 8 hours, and 37.2±3.6% (*p* < 0.01) at 24 hours. These results indicate a significant inhibition of IGF-1 synthesis in hepatocytes by GHRH(1-29)NH_2_ and its analog, MR-409.

Interestingly, the expression of IGF-2 was also decreased by GHRH(1-29)NH_2_ and MR-409 (Supplementary Figure 1B). In human hepatocytes treated by rhGH and GHRH(1-29)NH_2_, the expression of IGF-2 was reduced by 11.9±3.4% (*p* < 0.05) at 12 hours, and 25.8±5.9% (*p* < 0.05) at 24 hours, compared to the cells treated only with rhGH. In human hepatocytes treated with both rhGH and 1 µM MR-409, the reduction in mRNA for IGF-2 was 23.9±3.7% (*p* < 0.01) at 12 hours, 22.3±2.9% (*p* < 0.05) at 24 hours, and 40.0±9.4% (*p* < 0.01) at 48 hours. In general, the MR-409 induced a greater and more protracted inhibitory effect than GHRH(1-29)NH_2_. This may be linked to its much greater activity and the increased resistance to proteases of MR-409 (3), compared to GHRH(1-29)NH_2_.

### GHRH(1-29)NH_2_ and its agonist MR-409 inhibit synthesis and release of IGF-1 *in vitro* from rat hepatocytes

**Figure 3:**
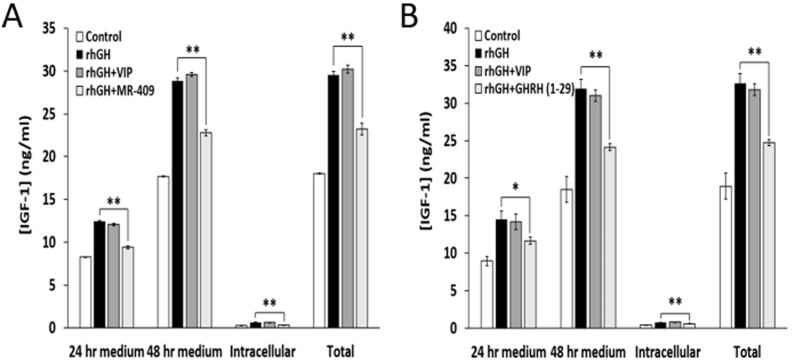
GHRH(1-29)NH_2_ and MR-409, but not VIP, inhibit synthesis and release of IGF-1 by rat hepatocytes Rat hepatocytes were treated by rhGH (1 µg/ml) alone or combined with **A.** 1 µM MR-409 or **B.** 1 µM GHRH(1-29)NH_2_. The concentrations of IGF-1 in the cell culture medium and in the cells were measured at 24 and 48 hours respectively by ELISA. Both MR-409 and GHRH(1-29)NH_2_ significantly inhibited the stimulatory effect of rhGH on the release of IGF-1. In both experiments (A) and (B), 1 µM VIP was tested as a peptide control and had no effect. (Error bars indicate ±SEM; *p < 0.05, **p < 0.01).

To test the effects of GHRH(1-29)NH_2_ and MR-409 on the synthesis and release of IGF-1 *in vitro*, rat hepatocytes were treated with 1 µg/ml of rhGH alone or in combination with 1 µM GHRH(1-29)NH_2_ or MR-409. As shown in Figure 3A, MR-409 not only inhibited the secretion of IGF-1 by the hepatocytes, but also decreased the concentration of IGF-1 in the cells. Thus, MR-409 significantly suppressed the release of IGF-1 induced by rhGH by 24.3±1.4% (*p* < 0.01) at 24 hours and by 21.1±1.2% (*p* < 0.01) at 48 hours. In addition, the intracellular IGF-1 level in the cells treated with MR-409 was 44.0±1.0% (*p* < 0.01) lower than in the cells exposed to rhGH alone. In a similar experiment using GHRH(1-29)NH_2_, rat hepatocytes treated with both rhGH (1 µg/ml) and 1 µM GHRH(1-29)NH_2_ secreted less IGF-1 compared to cells exposed only to rhGH. GHRH(1-29)NH_2_ significantly decreased the levels of IGF-1 in the medium by 19.6±3.3% (*p* < 0.05) at 24 hours and by 24.4±1.3% (*p* < 0.01) at 48 hours. GHRH(1-29)NH_2_ also lowered the intracellular concentration of IGF-1 by 23.1±1.1% (*p* < 0.01) (Figure 3B). Thus, both GHRH(1-29)NH_2_ and its agonist, MR-409, showed the capability to inhibit the synthesis and release of IGF-1 in rat hepatocytes treated with rhGH. In order to confirm the specificity of the inhibitory effects of GHRH(1-29)NH_2_ and MR-409 on the release of IGF-1, the vasoactive intestinal peptide (VIP), containing 28 amino acid residues, was used as a peptide control. There is a structural similarity between VIP and GHRH as well as considerable homology between their receptor proteins, which belong to the family of G protein coupled receptors. As shown in Figure 3A and 3B, VIP showed no effect on the IGF-1 secretion induced by rhGH in rat hepatocytes in contrast to GHRH(1-29)NH_2_ and GHRH agonist MR-409.

### GHRH(1-29)NH_2_ and MR-409 inhibit hepatic IGF-1 secretion in hypophysectomized rats

**Figure 4:**
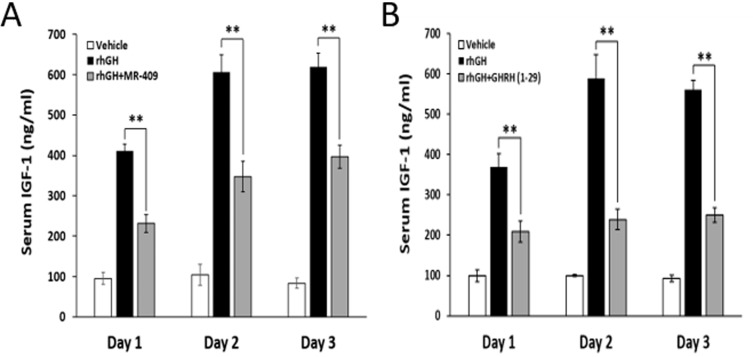
GHRH(1-29)NH_2_ and MR-409 suppress the hepatic IGF-1 secretion induced by rhGH Hypophysectomized rats (6 animals/group) were given daily i.p. injections of vehicle, rhGH alone or in combination with GHRH(1-29)NH_2_ or MR-409 for three continuous days. Group I, received only the vehicle; Group II, was injected with rhGH (0.5 mg/kg bw); Group III, received rhGH (0.5 mg/kg bw) plus either **A.** MR-409 (5 µg/25 g bw) or **B.** GHRH(1-29)NH_2_ (200 µg/25 g bw). Blood samples were collected at 24 hours after each injection. Serum IGF-1 was analyzed by ELISA. (Error bars indicate ±SEM; **p < 0.01).

In the week prior to the experiment, the baseline levels of IGF-1 in the blood of hypophysectomized rats were measured to be 58.8±8.5 ng/ml, indicating a successful elimination of the endocrine GH/IGF-1 axis. The animals were then treated daily for three days with rhGH, at a dose of 0.5 mg/kg bw, alone or together with the agonist MR-409, at 5 µg/25 g bw, as described in the legend to Figure 4. As shown in Figure 4A, rhGH strongly stimulated serum levels of IGF-1 immediately after the first injection, while MR-409 significantly inhibited this IGF-1 surge. The serum IGF-1 values were reduced by MR-409 from 410.6±17.4 ng/ml to 231.5±22.5 ng/ml, (*p* < 0.01), on day 1; from 605.8±43.9 ng/ml to 347.4±37.8 ng/ml, (*p* < 0.01), on day 2; and from 618.8±35.0 ng/ml to 396.4±28.6 ng/ml, (*p* < 0.01), on day 3. Compared to the effect of rhGH alone, the serum levels of IGF-1 in animals treated with both rhGH and MR-409 declined by 43.6±10.4% on day 1, 42.6±9.5% on day 2, and 36.0±7.8% on day 3. This result clearly demonstrates the inhibitory effect of MR-409 on the hepatic secretion of IGF-1 in response to the stimulus with GH.

In a parallel study, in which we used GHRH(1-29)NH_2_, at a high dose of 200 µg/25 g bw, about 40 times greater on the weight and molecular basis than that of MR-409, GHRH(1-29)NH_2_ diminished the rhGH-induced increase in IGF-1 levels by 43.3±7.1% (*p* < 0.01) on day 1, by 59.5±4.3% (*p* < 0.01) on day 2, and 55.4±3.2% (*p* < 0.01) on day 3 (Figure 4B). In our earlier pilot tests, GHRH(1-29)NH_2_ at dosages lower than 50 µg/25 g bw failed to significantly lower IGF-1 levels in animals treated with rhGH (0.5 mg/kg bw). This is likely due to a very short half-life of GHRH(1-29)NH_2_ in circulation [26].

### MR-409 suppresses serum IGF-1 in C57BL/6 mice

**Figure 5:**
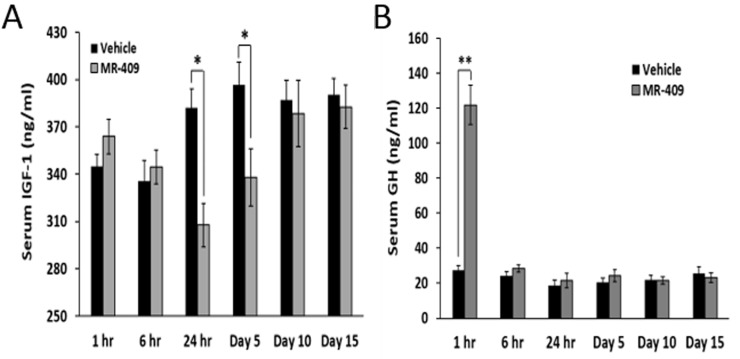
MR-409 suppresses the serum IGF-1 in C57BL/6 mice Wild type C57BL/6 mice (8 animals/group) were treated daily with either 5 μg/day MR-409 s.c. or vehicle for 15 days. Blood or serum samples were collected at selected times and used for **A.** IGF-1 and **B.** GH ELISAs. (Error bars indicate ±SEM; *p < 0.05, **p < 0.01).

The effect of MR-409 on serum IGF-1 was also tested in C57BL/6 mice. As shown in Figure 5A, daily injections of MR-409 significantly decreased serum IGF-1 from 24 hours on day 1 to day 5. At 24 hours, serum IGF-1 in mice treated with MR-409 was reduced from 382.1±12.1 ng/ml (in control mice) to 307.8±13.8 ng/ml (*p* < 0.05), and on day 5, from 396.5±14.4 ng/ml to 338.1±18.1 ng/ml, (*p* < 0.05). MR-409, however, showed a small stimulatory effect on serum IGF-1 within the first 6 hours after the injection (not significant). After day 10, the serum levels of IGF-1 in the animals treated with MR-409 became similar to those in controls. The same sera were also used to measure the concentrations of GH. Except for the first hour, no significant difference in GH levels was found between the animals treated with MR-409 and the control (Figure 5B).

### MR-409 down-regulates mRNA for IGF-1 in cancer cells

**Figure 6:**
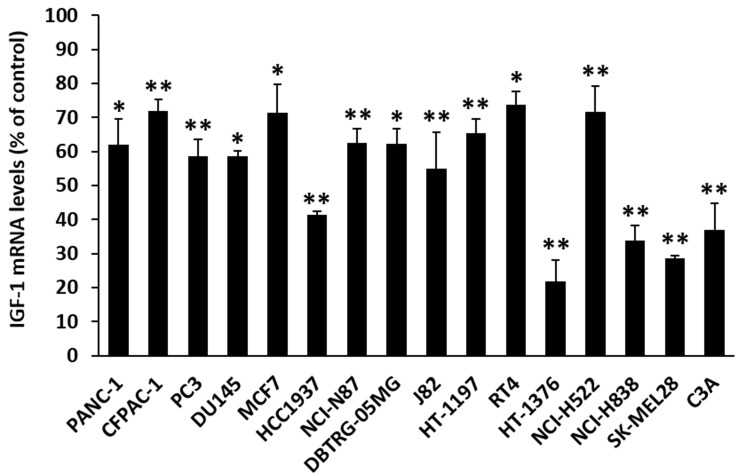
MR-409 down-regulates IGF-1 expression in cancer cells Cancer cells were treated with 10-1000 nM MR-409 in respective serum-reduced (1% FBS) medium for 48 hours. RNAs were isolated and analyzed by quantitative real-time PCRs. The inhibition of IGF-1 expression for each cancer cell was shown as % of the control (Error bars indicate ±SEM; *p < 0.05, **p < 0.01). (The definition of each cancer cell line is as follows; PANC-1, CFPAC-1: pancreatic cancer; PC3, DU145: prostate cancer; MCF7, HCC1937: breast cancer; NCI-N87: gastric cancer; DBTRG-05MG: glioblastoma; J82, HT-1197, RT4, HT-1376: bladder cancer; NCI-H522, NCI-H838, lung cancer; SK-MEL28: skin cancer, and C3A: liver cancer).

GHRH receptors are present in many tumors [1, 2, 27-33]. In order to determine whether GHRH agonist, MR-409, has an effect on the expression of IGF-1 in cancer cells, 16 human cancer cell lines were examined. The treatment of cancer cells consisted of *in vitro* exposure to concentrations of 10 nM to 1000 nM MR-409 for 48 hours as pre-determined by the cell proliferation assays. The mRNA levels for IGF-1 were analyzed at 12, 24, and 48 hours after the treatment. The effect on each cancer cell line is summarized in Figure 6. MR-409 exerted significant inhibitory effect on IGF-1 expression in all cancer cells tested. The mean mRNA level for IGF-1 was reduced by 46.0±16.5%, (*p* < 0.05) as compared to the control, the range being from 26.3±3.9% to 78.2±6.3%. Treatment of cancer cells with GHRH(1-29)NH_2_ also led to a decrease in mRNA levels for IGF-1. Thus GHRH(1-29)NH_2_ at 10 nM-1000 nM concentration reduced the mRNA for IGF-1 in J82, HT-1197, and HT-1376 bladder cancer cells by 28.3±9.5% (*p* > 0.05), 42.0±2.3% (*p* < 0.01), and 19.0±4.2% (*p* < 0.01), respectively; in CFPAC-1 and PANC-1 pancreatic cancer cells by 25.3±5.2% (*p* < 0.05) and 13.5±10.5% (*p* > 0.05), respectively; and in MCF7 breast cancer cells by 27.6±5.0% (*p* < 0.01).

### GHRH(1-29)NH_2_ and MR-409 inhibit the secretion of IGF-1 by cancer cells

We then determined the effects of GHRH(1-29)NH_2_ and its agonist, MR-409, on the secretion of IGF-1 by cancer cells *in vitro*. As summarized in Table 1, in both MCF7 breast cancer cells and J82 bladder cancer cells, the IGF-1 secretion was greatly stimulated by rhGH. The addition of MR-409 or GHRH(1-29)NH_2_ to culture medium induced a strong inhibition of the response to rhGH in both MCF7 and J82 cancer cells. In breast cancer lines, the stimulated level of rhGH (551.1 pg/ml) was reduced by 88.2±11.4% (*p* < 0.05) in the presence of MR-409 and by 70.4±14.2% (*p* < 0.05) after exposure to GHRH(1-29)NH_2_. In J82 bladder cancer cells, the stimulation induced by rhGH (125.2 pg/ml) was diminished by 64.6±24.4% (*p* > 0.05) in the presence of MR-409 and by 43.8±12.0% (*p* > 0.05) following the exposure to GHRH(1-29)NH_2_.

**Table 1:** Effect of GHRH(1-29)NH_2_ and its agonist MR-409 on the secretion of IGF-1 by cancer cells *in vitro*

Treatment		IGF-1 (pg/ml)^a^				
	MCF7 breast cancer			J82 bladder cancer		
	Measured level		Stimulation^b^	Measured level		Stimulation^b^
Control (no treatment)	529.9±53.5		-	240.3±24.7		-
rhGH (1µg/ml)	1081.0±113.1^*^		551.1	365.5±45.0		125.2
rhGH (1µg/ml) plus MR-409 (1 µM)	594.6±62.9^*^		64.7	284.6±30.5		44.3
rhGH (1µg/ml) plus GHRH(1-29) (1 µM)	692.7±78.4^*^		162.8	310.7±31.6		70.4

a. All values were normalized to the total protein in the dishes. The results are representive of three independent experiments.

b. Values were calculated against the value of the control.

Compared to the control, the measured IGF-1 level was significantly increased in the MCF7 cells treated with rhGH, *p < 0.05; MR-409 and GHRH(1-29) significantly decreased the IGF-1 secretion induced by rhGH, *p < 0.05.

### MR-356 suppresses serum IGF-1 in nude mice bearing NCI-N87 stomach cancer

**Figure 7:**
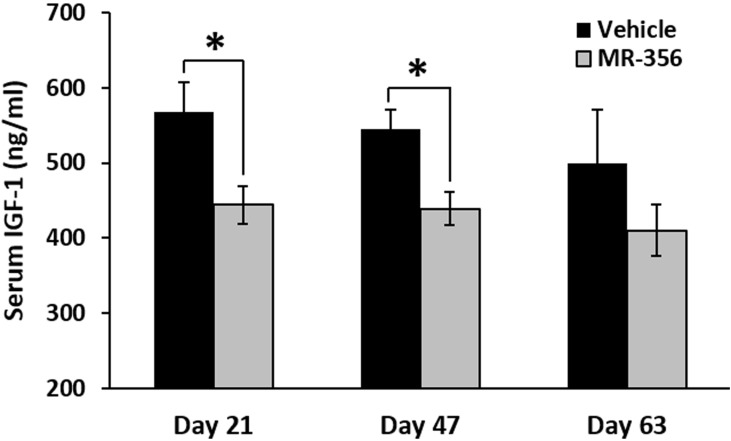
MR-356 suppresses serum IGF-1 in nude mice Nude mice were xenografted with NCI-N87 stomach cancer. For the treatment, animals were injected s.c. with 5 µg MR-356/day for 9 weeks. The blood from the control and treated groups (12 mice/group) were collected at selected days. Concentrations of serum IGF-1 were measured by ELISA. (Error bars indicate ±SEM; *p < 0.05).

Nude mice (12 animals per group) bearing xenografts of NCI-N87 stomach cancer, were treated with daily s.c. injections of 5 µg/day of MR-356, a potent agonistic analog of GHRH(1-29)NH_2_. Blood levels of IGF-1 were monitored throughout the treatment. As shown in Figure 7, serum levels of IGF-1 in the group treated with MR-356 were significantly lower than those of controls. The mean serum levels of IGF-1 in the treated group were reduced by 21.8±4.4%, (*p* < 0.05) on day 21, by 19.6±4.0%, (*p* < 0.05) on day 47, and by 17.9±9.2% (*p* > 0.05) on day 63. In addition, the treatment with MR-356 markedly decreased tumor growth as shown by the significant reduction of the mean volume of gastric tumors in the treated group to 360±111 mm^3^ on day 63 from that found in the control (1289±416 mm^3^) (*p* < 0.02).

### Effects of GHRH(1-29)NH_2_ and its analog, MR-409, on the GH-induced GHR/JAK2/STAT5 signaling

**Figure 8:**
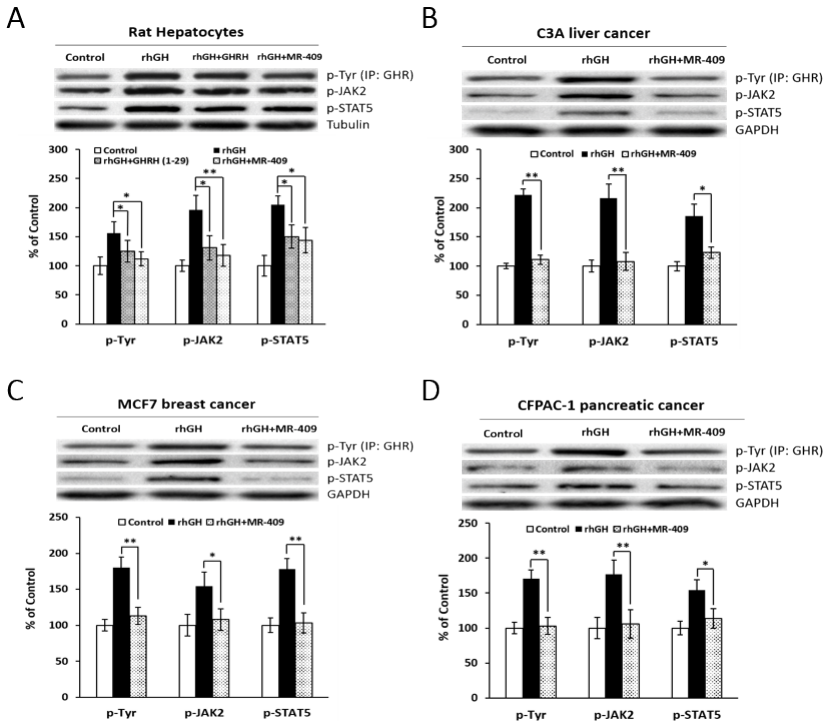
The effect of GHRH(1-29)NH_2_ and MR-409 on GHR/JAK2/STAT5 signaling **A.** Rat hepatocytes were treated by either 1 µg/ml rhGH alone or its combination with 1 µM GHRH(1-29)NH_2_ or MR-409 in serum-free William’s Medium E containing 0.1% BSA for 30 mins. The phosphorylation levels of GHR, JAK2, and STAT5 were analyzed by Western blots. **B.** C3A Human liver cancer cells, **C.** MCF7 breast cancer cells, and **D.** CFPAC-1 pancreatic cancer cells were treated by 1 µg/ml rhGH alone or a combination with 0.25 µM MR-409 for C3A and CFPAC-1, or 1 µM MR-409 for MCF7, in their respective serum-free basic medium for 30 mins. The protein levels for p-GHR, p-JAK2, and p-STAT5 were analyzed by Western blots. For quantification, expression levels of tubulin (for rat hepatocytes) or GAPDH (for human cancer cells) were used as references. (Error bars indicate ±SEM; *p < 0.05; **p < 0.01).

The GH receptor (GHR)-mediated JAK2/STAT5 pathway is a major mechanism, which regulates the IGF-1 production induced by GH [34], while members of the GH-inducible suppressor of cytokine signaling (SOCS) family and cytokine-inducible SH2 (CISH) proteins negatively regulate the GH-induced activation of JAK2/STAT5 signaling [35, 36]. Consequently, we investigated the role that GHRH(1-29)NH_2_ and its analog, MR-409, may play in these events. As shown in Figure 8A, both GHRH(1-29)NH_2_ and MR-409, reversed the activation of GHR/JAK2/STAT5 signaling in rat hepatocytes induced by GH. The phosphorylation levels of the signaling molecules were all significantly decreased (*p* < 0.05). Meanwhile, in the C3A human liver cancer cells, MCF-7 breast cancer cells and CFPAC-1 pancreatic cancer cells treated by MR-409, there was also a dramatic decrease in phosphorylation of GHR/JAK2/STAT5 (Figure 8B-8D). In addition, we evaluated the effects of GHRH(1-29)NH_2_ and MR-409 on the SOCSs and CISH. Treatment of human hepatocytes with GHRH(1-29)NH_2_ and MR-409 escalated the expression of SOCS1 by 192.6±19.7% (*p* < 0.05) and 206.3±17.6% (*p* < 0.01), respectively; mRNA for SOCS-2 by 122.4±22.5% (*p* > 0.05) and 146.9±29.9% (*p* < 0.05), respectively; and the expression of SOCS-3 by 156.9±2.3% (*p* < 0.05) and 196.9±24.4% (*p* < 0.05), respectively, compared to human hepatocytes exposed to rhGH. The expression of CISH, however, was not significantly changed (Supplementary Figure 2).

## DISCUSSION

Hypothalamic GHRH regulates the release of GH from the pituitary, and in turn GH stimulates the secretion of IGF-1 from the liver [1, 17, 25, 37-39]. The regulation of the secretion of IGF-1 is incompletely understood and appears to be mediated by multiple mechanisms [20, 23-25, 37, 40, 41]. Since IGF-1 is under the control of GH, the suppression of the release of GH from the pituitary by antagonistic analogs of GHRH disrupts the pituitary GH/hepatic IGF-1 axis and leads to a reduction in the blood levels of GH, which in turn results in a lowering of circulating IGF-1 [1, 2, 20, 24, 37, 42]. This inhibitory action of GHRH antagonists on IGF-1 levels in serum has been demonstrated by our group in nude mice bearing various human cancers [1, 27, 31, 43-46]. Nevertheless, the main inhibitory effect of GHRH antagonists on tumor growth appears to be exerted through GHRH receptors which are present in various human cancers [1, 2, 27-33].

In elaborate studies, Gahete, Kineman et al. and others documented that IGF-1 inhibits the release and synthesis of GH *in vitro* and *in vivo* by a feedback action [20]. However, the involvement of a neurohormonal component in control of IGF-1 release, has not been demonstrated to date. Recently, in the evaluations of the effects of our GHRH agonists on normal tissues and tumors, we have detected their inhibitory action on IGF-1 secretion. To the best of our knowledge, this phenomenon has not been previously reported. The demonstration of the inhibitory effects of GHRH agonists on the secretion of IGF-1, described herein, appears to be therefore a relevant contribution to knowledge on the regulation of GH/IGF-1 axis.

IGF-1 is a potent growth factor for various cancer cells [14-16, 18, 19, 21, 22, 24, 47-49]. Various experimental and clinical studies have demonstrated the role of IGF-1 in growth and metastasis of many cancers [16, 18, 19, 21, 22, 24]. The findings of epidemiological studies expertly reviewed by Samani et al [16] also indicate that IGF-1 is involved in the development of cancers. These clinical investigations have revealed that high levels of serum IGF-1 and/or decreased concentrations of IGF-1 binding proteins are linked to an augmented risk for several malignancies [16], including breast cancer [19, 50, 51], prostate [18, 52], lung [53], and colorectal cancer [54-56]. In our studies on GHRH antagonists, we have previously shown an association between the levels of serum or tumoral IGF-1 and tumor growth [27, 43-46]. We were aware that the reduction in the levels of IGF-1 was mostly induced by the inhibition of GH secretion by the GHRH antagonists. Our findings that GHRH(1-29)NH_2_ and its agonist, MR-409, can also reduce the secretion of hepatic and tumoral IGF-1 are unexpected. Nevertheless, the observations that the GHRH(1-29)NH_2_ and GHRH agonist, MR-409, inhibited the expression of mRNA for IGF-1 as well as IGF-1 secretion by human and rat hepatocytes and human cancer cells *in vitro*, after stimulation with rhGH, imply that GHRH agonists may exert these effects by direct action on the cells. The specificity of the effect of GHRH and its agonists was demonstrated by the fact that vasoactive intestinal peptide, structurally similar to GHRH, showed no effect. GHRH(1-29)NH_2_ and agonist MR-409 lowered serum IGF-1 levels in hypophysectomized rats injected with rhGH. These results and findings that GHRH agonists, MR-356 or MR-409, reduced serum IGF-1 levels in nude mice xenografted with human NCI-N87 stomach cancer as well as in C57BL/6 mice show that the inhibitory effects occur also *in vivo*. The inhibition of tumor growth which accompanied the treatment of NCI-N87 stomach cancer and other tumors by the GHRH agonists *in vivo* is the subject of our intense investigations, which will be reported elsewhere.

The mechanisms through which GHRH(1-29)NH_2_ and its agonist, MR-409, suppress IGF-1 production and release in hepatic and cancer cells is not completely understood. In this study, wild type C57BL/6 mice treated with MR-409 demonstrated a decrease in serum IGF-1 at 24 hours after the injection of the GHRH agonist. The GH levels, however, increased only within the first hour post injection and subsequently returned to the baseline levels 6 hours after injection. Thus, MR-409 showed only a transient stimulatory effect on GH release. These observations, suggest that in animals treated with GHRH agonists under these experimental conditions, serum IGF-1 may not be related to a fall in circulating GH levels. This action is different from that of GHRH antagonists which decrease the release of GH from pituitary somatotropes after binding to the GHRH receptors and block the production and release of GH [1, 37]. The decrease in circulating IGF-1 is then a consequence of blocking the secretion of GH as stated above. Such a mechanism, however, is not applicable to the GHRH agonists. Instead, the secretion of IGF-1 appears to be suppressed by a direct action of GHRH and its agonists on the hepatocytes and the tumors. This is supported by our studies using hepatocytes *in vitro* as well as hypophysectomized animals. In both cases, GHRH(1-29)NH_2_ and MR-409 suppressed the IGF-1 secretion stimulated by rhGH.

Incidentally, these are not the first findings of direct effects of GHRH agonists on extra-pituitary cells/tissues. GHRH(1-29)NH_2_ and its agonists have been demonstrated to exert direct stimulatory effects on cardiovascular endothelial cells [4-6], pancreatic β cells [7, 8], and fibroblasts [9]. These observations are in accord with direct effects of GHRH and its agonists on hepatocytes and cancer cells as found in our studies. In an effort to determine the mechanisms of action for these observations, we investigated whether the GH receptor (GHR)-mediated JAK2/STAT5 signaling transduction could be involved. In the course of stimulation with the GH, GHR/JAK2/STAT5 pathway plays a critical role in hepatic synthesis and release of IGF-1 [34, 57]. In cancer cells, this pathway was also found to be associated with tumoral expression of mRNA for IGF-1 [58] and the stimulation of proliferation [59] and survival of cancer cells [60]. We demonstrated that GHRH(1-29)NH_2_ and its agonist, MR-409, can suppress the GH-induced activation of GHR/JAK2/STAT5 pathway, which suggests that this mechanism of action is different from that of GHRH antagonists. Thus, the anti-neoplastic effect of GHRH antagonist, MIA-602, was found to be associated with blockage of GHRH-R-mediated PAK1/STAT3/NF-κB pathways in gastric cancers [28]. One possible explanation could be that the actions of GHRH agonists on the hepatocytes may involve a hypothetical unidentified factor, whose recruitment to the GH/GHR complex, after binding of GH to GHR, could be required for the activation of GHR and/or the maintenance of its kinase activity for downstream signaling. When GHRH or its agonists bind to the GHRH receptor, this leads to the dissociation of this unknown factor from the GHR, resulting in a quiescent complex with restrained capability of making transduction signals for the synthesis and release of IGF-1. This hypothesis, however, does not exclude a possibility that GHRH agonists may also bind to an unknown homologous G-protein coupled receptor and directly inhibit the IGF-1 production in both hepatic and tumor cells as proposed by Kineman for the actions of GHRH antagonists [37].

IGF-1 exerts its functions through the IGF-1 receptor (IGF1-R) [24]. In our preliminary studies on J82 bladder cancer, GHRH agonist MR-409 downregulated the expression of IGF1-R, inhibited the phosphorylation of IGF1-R and its downstream AKT and ERK pathways. In addition, GHRH(1-29)NH_2_ and MR-409 also induced an inhibition of mRNA for IGF-2 in human hepatocytes. An investigation on the effect of GHRH and its agonists on IGF-2 production may be also worth considering.

A major challenge to the use of GHRH *in vivo* has been its short half-life due to an enzymatic cleavage at the N-terminal [26]. To overcome this problem, various GHRH agonists, designated as the “MR” series, have been synthesized and evaluated by our group [3]. Some of these agonists, such as MR-409 and MR-356, showed significantly greater potency, higher binding affinities, and longer half-life *in vivo* due to increased resistance to degradation by proteases, compared to GHRH(1-29)NH_2_ [3]. Their effects on various cells/tissues have been reported in our previous publications [3-9, 61].

It is difficult to envision that GHRH could be involved in the pathophysiologic control of IGF-1 secretion since the circulating levels of hypothalamic GHRH are very low. In contrast, the levels of GHRH in the tumors can be very high [1, 38, 39]. The studies on this complex topic would require further extensive investigations. The results reported herein reveal the inhibitory effects of GHRH(1-29)NH_2_ and its agonists on the hepatic and tumoral secretion of IGF-1, which occurred under the conditions described.

## MATERIALS AND METHODS

### Peptide and chemicals

Synthesis and purification of MR-409 ((N-Me-Tyr^1^, D-Ala^2^, Orn^12,21^, Abu^15^, Nle^27^, Asp^28^)-GHRH(1-29)NHCH_3_) and MR-356 ((N-Me-Tyr^1^, Ala^2^, Phe^6^, Gln^8^, Orn^12^, Abu^15^, Orn^21^,Nle^27^, Asp^28^, Agm^29^)-GHRH(1-29)NH_2_) have been previously described [3]. Human GHRH(1-29)NH_2_ was supplied by AnaSpec (Fremont, CA). All other chemicals, if not specified, were purchased from Sigma-Aldrich (St. Louis, MO, USA).

### Experiments using hepatocytes

(I) Human and rat hepatocytes were treated with 0.5 µg/ml or 1 µg/ml rhGH respectively, or the combination of rhGH with GHRH(1-29)NH_2_ or its analog MR-409 (1 µM) in serum-free William’s Medium E containing 0.1% BSA. RNAs were isolated at selected times. The amounts of mRNA for target genes were analyzed by quantitative RT-PCR. (II) Rat hepatocytes (1×10^6^ cells/6-well) were treated with rhGH (1 µg/ml) alone or in combination with 1 µM GHRH(1-29)NH_2_ or MR-409. The culture media at 24 and 48 hours were collected for IGF-1 ELISA. Cell lysates at 48 hours were used for measurement of intracellular IGF-1 levels.

### IGF-1 secretion by cancer cells *in vitro*

Cancer cells (5×10^6^ cells per 10 cm culture dish) were pre-starved in serum-free medium for 6 hours, and then treated with 1 µg/ml rhGH alone or combined with 1 µM MR-409 or GHRH(1-29)NH_2_ for 48 hours. Cell culture media were then freeze-dried and reconstituted in distilled water. The salt was removed by using Amicon Ultra-2 column (Millipore). The levels of IGF-1 in the samples were measured by the Human IGF-1 Quantikine ELISA kit (R&D Systems).

### Animal studies *in vivo*

(I) Wild-type C57BL/6 mice (9 weeks old, 8 animals/group) were treated daily with 5 µg/day MR-409 s.c. or the vehicle for 15 days. Blood samples were collected from the tail vein at selected times. Serum IGF-1 and GH were determined by ELISAs. (II) Male Wistar Han IGS rats (Crl:WI), 6-weeks old, were hypophysectomized by Charles River Laboratories. Two weeks later, animals were then divided into three groups of six animals each, and were injected i.p. daily with rhGH at 0.5 mg/kg bw, or rhGH plus MR-409 at 5 µg/25 g bw, for three consecutive days. Control animals received the vehicle only. In a similar experimental setting, GHRH(1-29)NH_2_ (200 µg/25 g bw) was used instead of MR-409. At 24 hours after each injection, blood samples were collected for measurement of serum IGF-1. (III) Female athymic nude mice, bearing xenografted NCI-N87 human stomach cancers, were assigned to two groups of 12 mice each. Experimental group received 5 µg/day of MR-356 s.c. for 9 weeks and the control group the vehicle only (10% 1,2-propanediol). Tumor size was monitored on a weekly basis and blood samples were collected for measurement of IGF-1.

### Statistical analysis

Data are shown as mean ± SEM. To determine statistical significance, one-way ANOVA followed by unpaired two-tailed Student’s *t*-test were used (Origin v6.0). Differences were considered significant if *p* < 0.05.

More detailed information on materials and methods is provided in Supplementary Materials.

## SUPPLEMENTARY MATERIALS FIGURES AND TABLE


