# Supplementary material for: Growth hormone-releasing hormone (GHRH) and its agonists inhibit hepatic and tumoral secretion of IGF-1
Source: Oncotarget. 2018 Jun 19;9(47):28745–56. doi: 10.18632/oncotarget.25676 (PMC6033336; doi:10.18632/oncotarget.25676)
Supplement: Supplementary file 1 [file oncotarget-09-28745-s001.pdf]

Growth hormone releasing hormone (GHRH) and its agonists inhibit hepatic and tumoral secretion of IGF-1

SUPPLEMENTARY MATERIALS

Cell culture

All cancer cell lines were obtained from the American Type Culture Collection (ATCC). Cells were grown and maintained as instructed.

Quantitative RT-PCR

All RNAs were isolated by RNeasy Mini kit (Qiagen, Valencia, CA). 1 µg of total RNA was reversely transcribed into cDNA using iScript cDNA Synthesis kit (Bio-Rad, Hercules, CA). For quantitative real-time PCRs, 0.05-5 ng cDNAs and 0.5 µM of each primer in 20 µl of 1× iQ SYBR Green Supermix (Bio-Rad, Hercules, CA) were amplified using CFX96 Touch Real-Time PCR Detection System (Bio-Rad, Hercules, CA). The PCR program included one cycle of 3 minutes at 95°C; followed by 40 cycles of 15 seconds at 95°C and 30 seconds at 62°C. Absence of non-specific PCR products was confirmed by melting the PCR products at 0.5°C/minute from 65°C to 95°C. All reactions were done in triplicate, and gene expression analysis was conducted using CFX Manager software (Bio-Rad, Hercules, CA). All PCR primers used for RT-PCR are listed in Supplementary Table 1.

IP (Immunoprecipitation)

For immunoprecipitation, 1 mg total protein was incubated with a mouse monoclonal antibody against GHR (Santa Cruz Biotechnology) overnight at 4 °C. Protein G Sepharose 4B (ThermoFisher) was then added and the mixture was incubated for an additional 4 hours at 4 °C. The beads were then washed three times in ice-cold cell lysis buffer. Immunoprecipitated proteins were eluted by 2× Laemmli sample buffer and used for Western blotting against p-Tyr (clone 4G10, Millipore).

Western blots

Cells were washed in cold PBS and lysed in cell lysis buffer (50 mM Tris pH 7.5, 150 mM NaCl, 1% Triton X-100, 10% glycerol, 0.5 nM EDTA) supplemented with fresh 1 mM PMSF (phenylmethanesulfonylfluoride) and protease inhibitor cocktail (Sigma-Aldrich, St. Louis, MO). Protein extract from the cell lysate was electrophoresed on 4-12% SDS-PAGE gels and then transferred to an Immobilon-P PVDF membrane (Millipore, Billerica, MA). The membrane was blocked in 5% BSA, incubated with specific primary antibodies at 4°C overnight, followed by a 1 hour incubation with secondary antibodies at room temperature. Bands were detected using ECL detection reagents, followed by exposure to ECL Hyperfilm (GE Healthcare, Piscataway, NJ). For quantitative analysis, films were photographed using a Bio-Rad Gel Documentation System (Bio-Rad, Hercules, CA). The intensity of each specific band was measured using a Quantity One software (Bio-Rad, Hercules, CA). The primary antibodies used were: GHRH-R (ab76263, Abcam); GAPDH (sc-47724, Santa Cruz Biotechnology); p-JAK2 (#3776), p-STAT5 (#9351), and α-Tubulin (#3873) (Cell Signaling Technology).

Experiments using hepatocytes

Experiment 1 (Gene expression study): Human and rat (Sprague-Dawley, male) plateable hepatocytes were purchased from ThermoFisher Scientific and handled as instructed. After thawing, cells were plated in a collagen I-coated 6-well plates at a density of 1×10^6 cell/well. Cells were allowed to attach in the Plating Medium (William's Medium E with 5% FBS, cocktail A, and 1 µM dexamethasone) for 6 hours. The medium was then replaced by the Maintenance Medium (William's Medium E with cocktail B and 0.1 µM dexamethasone). After being kept in the Maintenance Medium for 24 hours, in a 5% CO_2 incubator at 37°C, cells were starved overnight in serum-free William's Medium E supplemented with 0.1 µM dexamethasone, 2 mM L-glutamine, 100 U/ml penicillin and 100 µg/ml streptomycin. The next day, human hepatocytes were treated with either rhGH (0.5 µg/ml) or rhGH (0.5 µg/ml) in combination with GHRH(1-29)NH_2 or its analog MR-409 (1 µM), in serum-free William's Medium E containing 0.1% BSA. rhGH was added simultaneously with GHRH(1-29)NH_2 or MR-409. The rat cells were treated with either rhGH (1 µg/ml) alone or rhGH (1 µg/ml) together with GHRH(1-29)NH_2 or MR-409 (1 µM), respectively. Control cells were treated in the same medium containing the peptide dissolving buffer (0.5% DMSO in PBS). RNAs were isolated at selected times. The amount of mRNA for target genes was analyzed by quantitative RT-PCR.

Experiment 2 (IGF-1 synthesis and release *in vitro*): Rat hepatocytes were plated and maintained in 6-well plates as described above. Hepatocytes were treated with either rhGH (1 µg/ml) itself or in presence of 1 µM GHRH(1-29)NH_2 or MR-409. The culture media were collected at 24 and 48 hours for IGF-1 ELISA (Mouse/Rat IGF-I Quantikine ELISA Kit, R & D Systems). At the end of the experiment, cells were harvested by trypsinization, washed twice in cold PBS, and eventually resuspended in 0.35 ml cold PBS. Subsequently, cells were lysed by repetitive freezing/thawing, homogenized by passing through 25-gauge needles, and were further subjected to sonication. The cell lysate was then centrifuged at 14,000 rpm for 15 min at 4°C. The supernatant was collected for measurement of intracellular IGF-1, and also for quantification of total protein (BCA Protein Assay Kit, Pierce).

IGF-1 secretion by cancer cells *in vitro*

For the measurement of IGF-1 secretion by cancer cells, 5×10^6 cells were seeded in a 10 cm cell culture dish and allowed to attach for 24 hours. Cells were subsequently starved in serum-free medium for 6 hours and then treated with 1 µg/ml rhGH alone or combined with 1 µM MR-409 or GHRH(1-29)NH_2. After 48 hours of incubation, the cell culture medium was lyophilized and resuspended in 1 ml sterile water. The salt in the concentrated media were removed by using Amicon Ultra-2 column (Millipore). The levels of IGF-1 in the prepared samples were measured by the Human IGF-1 Quantikine ELISA kit (R&D Systems). For the calculation, the IGF-1 concentration for each sample was normalized to the total protein in the dish.

Signaling pathway studies

Rat plateable hepatocytes were plated and maintained as described above. The day before the experiment, cells were starved in serum-free William's Medium E overnight. Cells were then treated by either 1 µg/ml rhGH or its combination with 1 µM MR-409, or GHRH(1-29)NH_2, in serum-free William's Medium E containing 0.1% BSA. In the experiments using cancer cells, cells were starved in serum-free basic medium overnight. The next day, cells were treated by either 1 µg/ml rhGH alone or its combination with MR-409 at the doses determined by their respective cell proliferation assay. In both cases, cell lysates were harvested at 15, 30 and 60 min after the treatment and used for immunoprecipitation and Western blots.

Animal studies *in vivo*

In the first experiment, wild-type C57BL/6 mice, 9 weeks old were purchased from the Charles River Laboratories. Animals received water and standard laboratory chow diet throughout the whole experiment and were allowed 1 week to acclimatize to their new environment. At week 10, animals (8 mice/group) were treated daily with either 5 µg/day MR-409 s.c. or the vehicle for 15 days. Blood samples were collected from

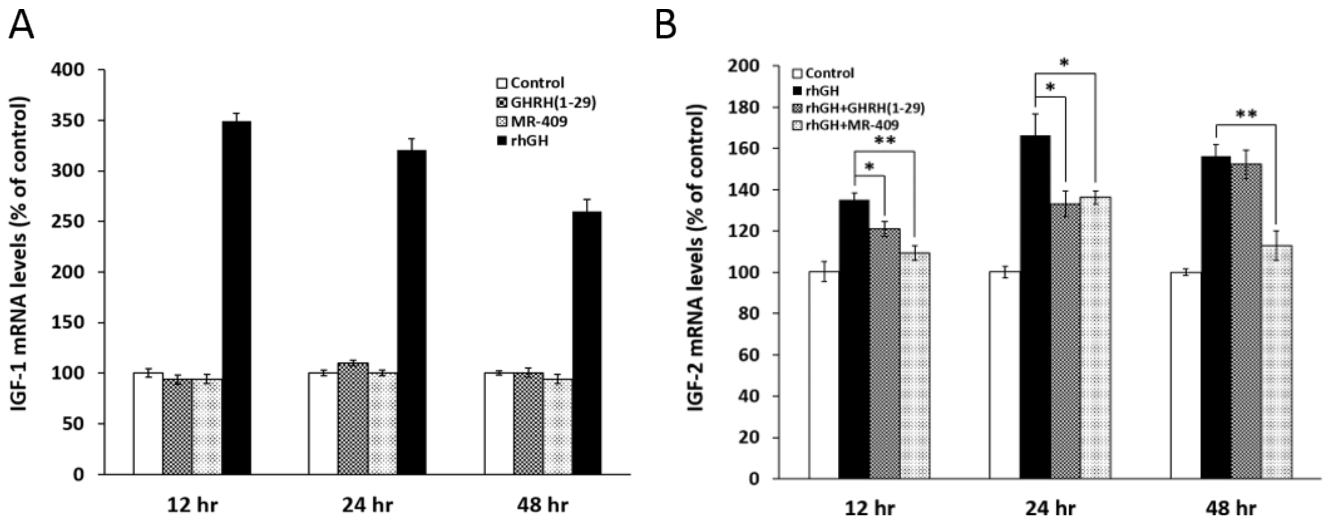

Supplementary Figure 1: The effect of GHRH(1-29)NH_2 and MR-409 on the expression of IGF-1 and IGF-2 in human hepatocytes. A. Human hepatocytes were treated with either 0.5 µg/ml rhGH or 1 µM GHRH(1-29)NH_2 or 1 µM MR-409 for selected times. The RNAs were isolated and the levels of mRNA for IGF-1 were determined by quantitative RT-PCR. **B.** Human hepatocytes were treated with either rhGH alone (0.5 µg/ml) or a combination of rhGH with 1 µM GHRH(1-29)NH_2 or MR-409. The RNAs were isolated at selected times and the levels of mRNA for IGF-2 were determined by quantitative RT-PCR. (Error bars indicate ±SEM; *$p < 0.05$; **$p < 0.01$).

Supplementary Table 1: Primers used for quantitative real-time PCRs

Target	Forward primer	Reverse primer
h IGF-1	TTCAACAAGCCCACAGGGTAT	CTCCGGAAGCAGCACTCATC
h IGF-2	GGCGGCATTTGGGATACA	TCTGTCATGGTGGAAAGATGGA
h SOCS-1	GCTCCTTCCCCTTCCAGATT	CCACATGGTTCCAGGCAAGT
h SOCS-2	TCAAAGGTCCAGGCTCCAGTA	GATCAGCAGGCATATCAAATTGC
h SOCS-3	CCTGGTGGGACGATAGCAA	TGGCAGTTCTCATTAGTTCAGCAT
h CISH	CTGTGCATAGCCAAGACCTTCTC	AAGCTGGAGTCGGCATACTCA
h GAPDH	ACCCACTCCTCCACCTTTGAC	CCCTGTTGCTGTAGCCAAATTC
r IGF-1	ACAGGCTATGGCTCCAGCAT	CTCCAGCCTCCTCAGATCACA
r GAPDH	CTCCCATTCTTCCACCTTTGAT	CCCTGTTGCTGTAGCCATATTC

the tail veins at the selected times. Sera were prepared and used for measuring the concentrations of IGF-1 and GH by ELISA (Mouse/Rat IGF-I Quantikine ELISA Kit, R & D Systems; Growth Hormone (GH) ELISA Kit, Bertin Pharma).

In the second experiment, male Wistar Han IGS rats (Crl:WI), 6-weeks old, were hypophysectomized by the supplier (Charles River Laboratories). Animals were fed with standard laboratory chow diet and water containing 5% sucrose. Body weights were monitored every week after arrival. Animals with a weight gain less than 3 g/week were considered as hypophysectomized (hypox). Animals were allowed to adapt to their environment for one week. Three baseline blood samples were then collected in the week before the experiment. When hypox rats reached an age of 8 weeks, they were randomly divided into three groups. Six animals in each group were then injected (i.p.) daily with either rhGH (0.5 mg/kg bw), or rhGH (0.5 mg/kg bw) plus MR-409 (5 µg/25 g bw), for three consecutive days. Control animals received the vehicle only. In a repetitive experiment setting, the MR-409 was replaced by GHRH(1-29)NH_2 (200 µg/25 g bw). At 24 hours after each injection, blood samples were collected from the tail veins and a following daily injection was performed. Sera were prepared and used for measurement of IGF-1. For all injections, rhGH was purchased from the National Hormone & Peptide Program at Harbor-UCLA Medical Center, and resuspended in saline immediately before use. MR-409 was dissolved in DMSO and diluted in 10% propylene glycol (Sigma-Aldrich).

In the third experiment, female athymic nude mice, 5-6 weeks old, obtained from the Charles River Laboratories, were housed in a temperature and humidity controlled facility at a 12hr light/12hr dark schedule. Animals were fed standard laboratory diet and water. The donors for tumor xenograft were generated as previously described. Subsequently, the tumors harvested from the donor were xenografted subcutaneously into both flanks of each animal. Tumors were allowed to grow to a mean volume of 100 mm^3 prior to the initial treatment. Animals were randomly assigned to two groups (6 mice/group). In the treated group, animals were injected 5 µg/day of MR-

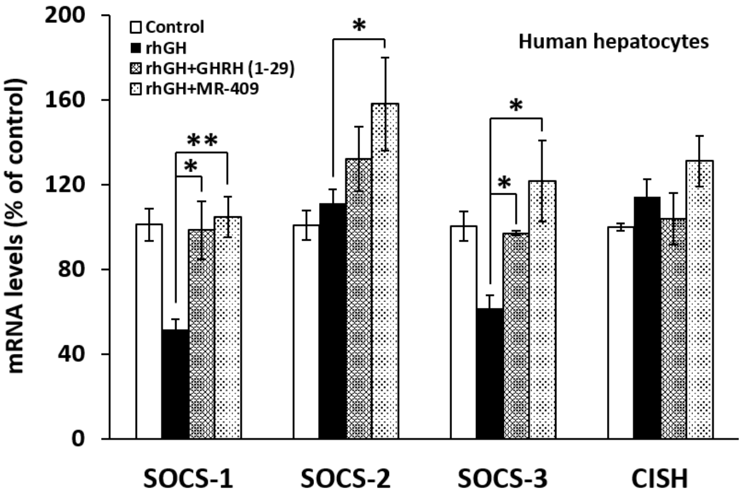

Supplementary Figure 2: The effect of GHRH(1-29)NH_2 and MR-409 on the expression of inhibitors of GHR/JAK2/STAT5 signaling. Human hepatocytes were treated by either 0.5 µg/ml rhGH alone or its combination with 1 µM GHRH(1-29)NH_2 or MR-409 in serum-free William's Medium E containing 0.1% BSA for 24-48 hours. The mRNA levels for SOCSs and CISH were measured by quantitative RT-PCR (Error bars indicate ±SEM; *$p < 0.05$; **$p < 0.01$).

356 s.c. for 9 weeks. The control animals received (10% 1,2-propanediol) vehicle only. Tumor size was monitored on a weekly basis. Blood samples during the treatment were also collected for measurement of IGF-1.

All animal procedures were approved by the Veterans Affairs Animal Care and Use Committee and were conducted in accordance with additional institutional guidelines as well.
